# Supplementary material for: Admission systolic blood pressure as a prognostic predictor of acute decompensated heart failure: A report from the KCHF registry
Source: PLoS One. 2021 Jul 2;16(7):e0253999. doi: 10.1371/journal.pone.0253999 (PMC8253441; doi:10.1371/journal.pone.0253999)
Supplement: S1 Fig — (A) All-cause death, (B) Cardiovascular death, (C) Noncardiovascular death, and (D) Hospitalization for HF. Follow-up was commenced on the day of discharge. SBP = systolic blood pressure, HF = heart failure. (PDF) [file pone.0253999.s002.pdf]

S1 Fig. Kaplan-Meier curves for postdischarge clinical events based on further subdivided range of blood pressure (<100, 100–119, 120–139, 140–159, and ≥160 mmHg).

(A) All-cause death, (B) Cardiovascular death, (C) Noncardiovascular death, and (D) Hospitalization for HF. Follow-up was commenced on the day of discharge.

SBP = systolic blood pressure, HF = heart failure.

(A) All-cause Death

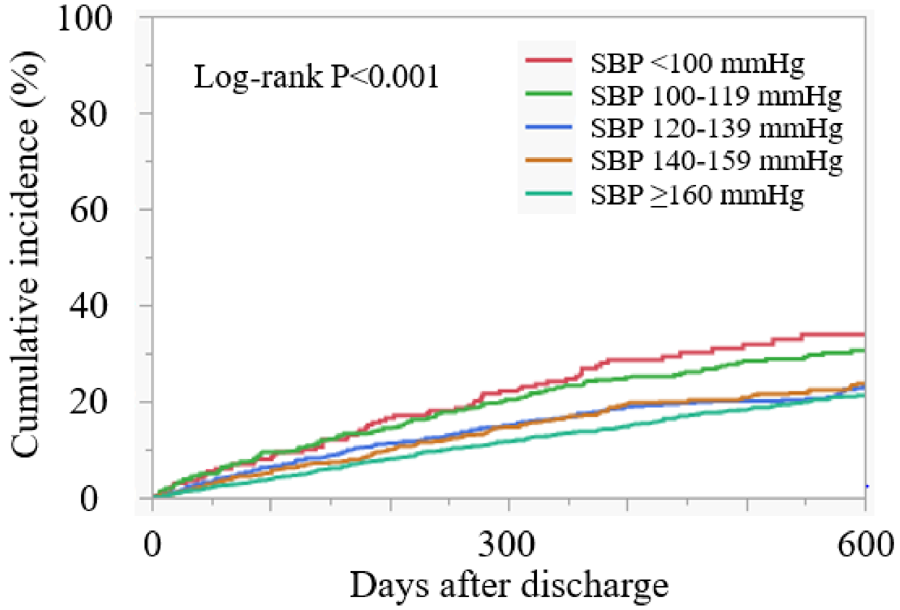

N of patients at risk	0 days	30 days	90 days	180 days	1 year
SBP <100 mmHg	206	195	185	170	135
SBP 100-119 mmHg	532	506	474	444	346
SBP 120-139 mmHg	777	751	715	677	547
SBP 140-159 mmHg	811	786	753	718	557
SBP ≥160 mmHg	1238	1193	1157	1097	934

(B) Cardiovascular Death

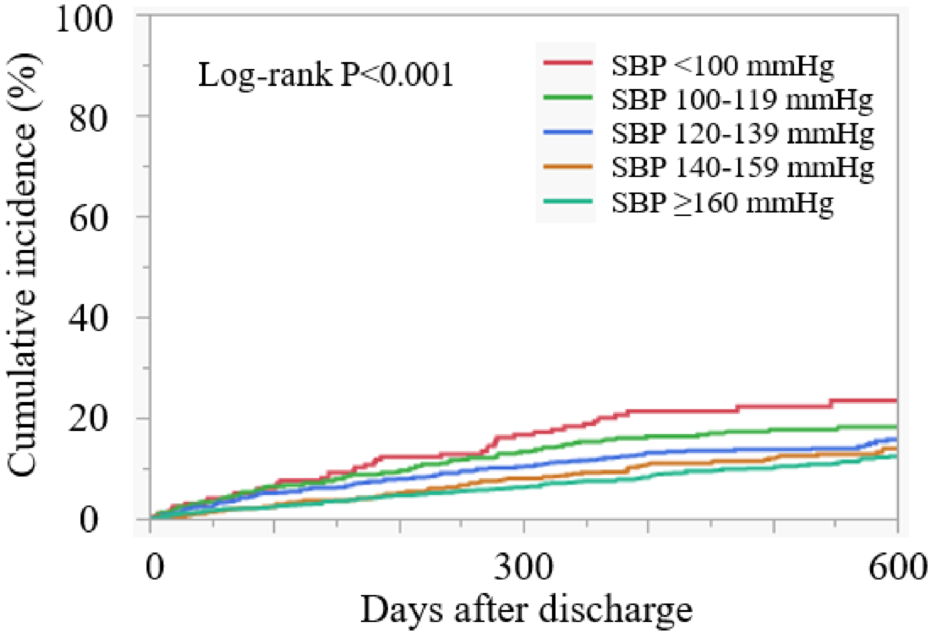

N of patients at risk	0 days	30 days	90 days	180 days	1 year
SBP <100 mmHg	206	195	185	170	135
SBP 100-119 mmHg	532	506	474	444	346
SBP 120-139 mmHg	777	751	715	677	547
SBP 140-159 mmHg	811	786	753	718	557
SBP ≥160 mmHg	1238	1193	1157	1097	934

(C) Non-cardiovascular Death

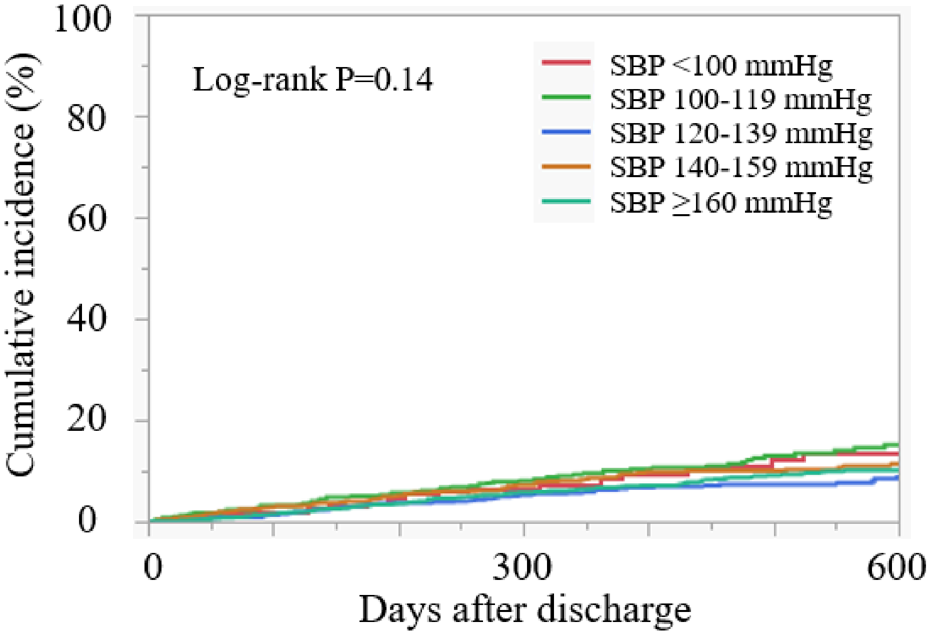

N of patients at risk	0 days	30 days	90 days	180 days	1 year
SBP <100 mmHg	206	195	185	170	135
SBP 100-119 mmHg	532	506	474	444	346
SBP 120-139 mmHg	777	751	715	677	547
SBP 140-159 mmHg	811	786	753	718	557
SBP ≥160 mmHg	1238	1193	1157	1097	934

(D) Hospitalization for HF

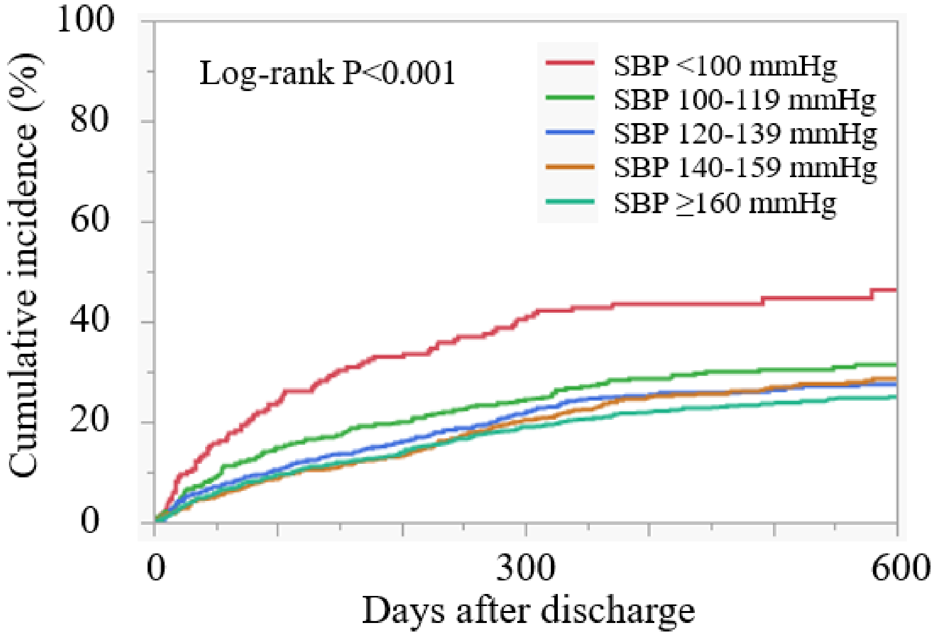

N of patients at risk	0 days	30 days	90 days	180 days	1 year
SBP <100 mmHg	206	178	150	124	88
SBP 100-119 mmHg	532	475	418	377	271
SBP 120-139 mmHg	777	713	659	591	435
SBP 140-159 mmHg	811	759	694	640	443
SBP ≥160 mmHg	1238	1152	1067	980	764
