# Supplementary material for: Admission systolic blood pressure as a prognostic predictor of acute decompensated heart failure: A report from the KCHF registry
Source: PLoS One. 2021 Jul 2;16(7):e0253999. doi: 10.1371/journal.pone.0253999 (PMC8253441; doi:10.1371/journal.pone.0253999)
Supplement: S1 Table — (PDF) [file pone.0253999.s003.pdf]

S1 Table. Patient Characteristics on Admission.

Variables	Entire cohort (N=3804)	Admission SBP <100 mmHg (N=253)	Admission SBP 100–139 mmHg (N=1411)	Admission SBP ≥140 mmHg (N=2140)	P value
Age, years	78.1 ± 12.0	75.4 ± 14.4	78.0 ± 12.1	78.5 ± 11.7	0.02
≥80 years [a]	2040 (54)	116 (46)	766 (54)	1158 (54)	0.04
Men [a]	2077 (55)	146 (58)	798 (57)	1133 (53)	0.06
Body mass index, kg/m^2 [b]	22.8 ± 4.5	22.0 ± 4.3	22.7 ± 4.4	22.9 ± 4.5	0.003
<22 kg/m^2 [a]	1706 (47)	137 (58)	638 (47)	931 (46)	0.003
Prior hospitalization for heart failure [a]	1402 (38)	136 (56)	588 (42)	678 (32)	<0.001
Number of hospitalization due to heart failure	1 (1–2)	2 (1–4)	1 (1–2)	1 (1–2)	<0.001
Current smoker [a]	428 (11)	16 (6.6)	137 (10)	275 (13)	<0.001
Ambulatory [a]	2938 (78)	188 (76)	1091 (78)	1659 (78)	0.60
Living alone [a]	813 (21)	37 (15)	297 (21)	479 (22)	0.02
**Etiology**					<0.001
Coronary artery disease	1085 (29)	71 (28)	405 (29)	609 (28)	
Cardiomyopathy	604 (16)	84 (33)	284 (20)	236 (11)	
Hypertensive heart disease	984 (26)	15 (5.9)	191 (14)	778 (36)	
Valvular heart disease	818 (22)	49 (19)	380 (27)	389 (18)	
Others	313 (8)	34 (13)	151 (11)	128 (6)	
**Concomitant diseases**					
Hypertension [a]	2725 (72)	123 (49)	909 (64)	1693 (79)	<0.001
Dyslipidemia	1424 (37)	80 (32)	529 (37)	815 (38)	0.13

Diabetes [a]	1381 (36)	83 (33)	480 (34)	818 (38)	0.02
Prior myocardial infarction [a]	845 (22)	56 (22)	300 (21)	489 (23)	0.54
Prior stroke [a]	623 (16)	40 (16)	228 (16)	355 (17)	0.91
Atrial fibrillation or flutter [a]	1643 (43)	131 (52)	723 (51)	789 (37)	<0.001
Ventricular tachycardia or fibrillation	159 (4.2)	33 (13)	73 (5.2)	53 (2.5)	<0.001
Malignant neoplasm	550 (14)	32 (13)	187 (13)	331 (15)	0.13
Chronic lung disease [a]	517 (14)	25 (9.9)	193 (14)	299 (14)	0.20
Prior percutaneous coronary intervention	813 (21)	49 (19)	287 (20)	477 (22)	0.28
Prior coronary artery bypass grafting	279 (7.3)	22 (8.7)	112 (7.9)	145 (6.8)	0.30
**Hemodynamic data on admission**					
Heart rate, bpm	95.8 ± 27.5	89.2 ± 26.2	93.4 ± 26.8	98.2 ± 27.9	<0.001
<60 bpm [a]	246 (6.5)	25 (10)	89 (6.4)	132 (6.2)	0.067
Systolic blood pressure, mmHg	147.6 ± 35.2	88.2 ± 9.2	121.8 ± 10.9	171.7 ± 25.9	<0.001
Diastolic blood pressure, mmHg	84.5 ± 24.0	56.5 ± 10.4	73.8 ± 14.5	95.0 ± 24.4	<0.001
**Symptoms on admission**					
NYHA class 3 or 4	3314 (88)	224 (89)	1189 (85)	1901 (89)	<0.001
Orthopnea	2979 (81)	188 (78)	1035 (76)	1756 (84)	<0.001
Rales	2915 (79)	174 (71)	1011 (74)	1730 (83)	<0.001
Dyspnea on exertion	3746 (95)	228 (96)	1290 (95)	1958 (95)	0.75
Jugular venous distention	2830 (79)	176 (74)	1031 (78)	1623 (80)	0.04
Peripheral edema	2903 (78)	177 (72)	1100 (80)	1626 (78)	0.008
**Chest radiograph on admission**					
Pulmonary congestion	3499 (93)	222 (89)	1257 (90)	2020 (95)	<0.001

Pleural effusion	3288 (87)	206 (82)	1233 (88)	1849 (87)	0.04
**Laboratory values on admission**					
Hemoglobin, mg/dL	11.5 ± 2.3	11.3 ± 2.3	11.4 ± 2.3	11.5 ± 2.3	0.44
Anemia [a,c]	2575 (68)	181 (72)	955 (68)	1439 (67)	0.36
Serum creatinine, mg/dL	1.11 (0.83–1.64)	1.43 (0.99–2.13)	1.11 (0.83–1.62)	1.08 (0.81–1.61)	<0.001
eGFR, mL/min/1.73 m^2	43.6 (28.2–60.2)	33.9 (22.3–49.8)	43.3 (28.7–60.4)	44.9 (28.9–60.8)	<0.001
<30 mL/min/1.73m^2 [a]	1051 (28)	104 (41)	381 (27)	566 (26)	<0.001
Albumin, g/dL	3.5 ± 0.5	3.3 ± 0.5	3.4 ± 0.5	3.5 ± 0.5	<0.001
<3 g/dL [a]	533 (14)	49 (20)	203 (15)	281 (13)	0.012
Serum sodium, mmol/L	139 ± 4.3	137 ± 5.4	139 ± 4.3	139 ± 4.1	<0.001
<135 mmol/L [a]	481 (13)	62 (25)	189 (13)	230 (11)	<0.001
Serum potassium, mmol/L	4.2 ± 0.7	4.4 ± 0.8	4.2 ± 0.7	4.2 ± 0.7	<0.001
Total bilirubin, mg/dL	0.7 (0.5–1.1)	0.9 (0.6–1.3)	0.8 (0.6–1.2)	0.7 (0.5–1.0)	<0.001
Brain-type natriuretic peptide, pg/mL	722 (403–1294)	874 (446–1540)	707 (404–1245)	721 (397–1286)	0.038
**Echocardiographic parameters**					
Left ventricular ejection fraction, %	46 (33–60)	40 (24–56)	44 (31–60)	48 (35–60)	<0.001
<40% [a]	1437 (38)	126 (50)	583 (41)	728 (34)	
40%–49%	686 (18)	37 (15)	229 (16)	420 (20)	
≥50%	1667 (44)	90 (36)	596 (42)	981 (46)	
Moderate–severe mitral regurgitation	1208 (34)	89 (41)	531 (41)	588 (30)	<0.001
Moderate–severe aortic stenosis	234 (6.7)	15 (6.8)	97 (7.5)	122 (6.2)	0.33
**Oral medications on admission**					
β-blocker	1506 (40)	151 (60)	577 (41)	778 (36)	<0.001

Mineralocorticoid receptor antagonist	718 (19)	89 (35)	338 (24)	291 (14)	<0.001
ACEI or ARB	1736 (46)	111 (44)	612 (43)	1013 (47)	0.06
Loop diuretics	1919 (50)	184 (73)	844 (60)	891 (42)	<0.001
Thiazide	245 (6.4)	22 (8.7)	111 (7.9)	112 (5.2)	0.002
Tolvaptan	165 (4.3)	37 (15)	82 (5.8)	46 (2.2)	<0.001
Calcium channel blocker	1382 (36)	58 (23)	497 (35)	827 (39)	<0.001

Values are number (%), mean ± SD, or median (interquartile range).

SBP=systolic blood pressure; NYHA=New York Heart Association; eGFR=estimated glomerular filtration rate; ACEI=angiotensin-converting enzyme inhibitor; ARB=angiotensin II receptor blocker.

[a] Risk-adjusting variables selected in the logistic regression models for all-cause death, cardiovascular death, and non-cardiovascular death during hospitalization.

[b] Body mass index was calculated as weight in kilograms divided by height in meters squared.

[c] Anemia was defined by the World Health Organization criteria (hemoglobin <12.0 g/dL in women and <13.0 g/dL in men).
