# Supplementary material for: Admission systolic blood pressure as a prognostic predictor of acute decompensated heart failure: A report from the KCHF registry
Source: PLoS One. 2021 Jul 2;16(7):e0253999. doi: 10.1371/journal.pone.0253999 (PMC8253441; doi:10.1371/journal.pone.0253999)
Supplement: S2 Table — (PDF) [file pone.0253999.s004.pdf]

S2 Table. In-hospital Management.

Variables	Entire cohort (N=3804)	Admission SBP <100 mmHg (N=253)	Admission SBP 100-139 mmHg (N= 1411)	Admission SBP ≥140 mmHg (N=2140)	P value
**Management in the emergency room**					
Respiratory management	2768 (73)	157 (62)	909 (64)	1702 (80)	<0.001
Oxygen inhalation	2203 (58)	136 (54)	809 (57)	1258 (59)	0.27
Non-invasive positive pressure ventilation	511 (13)	15 (5.9)	91 (6.5)	405 (19)	<0.001
Invasive positive pressure ventilation	54 (1.4)	6 (2.4)	9 (0.6)	39 (1.8)	0.006
Intravenous drugs					
Furosemide	1959 (52)	84 (33)	672 (48)	1203 (56)	<0.001
Nitroglycerin/isosorbide dinitrate	683 (18)	1 (0.4)	35 (2.5)	647 (30)	<0.001
Dopamine	16 (0.4)	4 (1.6)	9 (0.6)	3 (0.1)	0.001
Dobutamine	97 (2.6)	19 (7.5)	45 (3.2)	33 (1.5)	<0.001
Norepinephrine	30 (0.8)	11 (4.4)	9 (0.6)	10 (0.5)	<0.001
**Intravenous drugs ≤24 hours after hospital presentation**					
Furosemide	3229 (85)	193 (76)	1231 (87)	1805 (84)	<0.001
Nitroglycerin/isosorbide dinitrate	916 (24)	6 (2.4)	80 (5.8)	830 (39)	<0.001
Nicardipine	242 (6.4)	3 (1.2)	8 (0.6)	231 (11)	<0.001
Carperitide	1409 (37)	51 (20)	457 (32)	901 (42)	<0.001
Dopamine	72 (1.9)	18 (7.1)	30 (2.1)	24 (1.1)	<0.001
Dobutamine	449 (12)	84 (33)	225 (16)	140 (6.5)	<0.001
Norepinephrine	83 (2.2)	30 (12)	26 (1.8)	27 (1.3)	<0.001

**In-hospital mechanical support**					
Respiratory tract					
Noninvasive positive pressure ventilation	841 (23)	54 (22)	189 (14)	598 (28)	<0.001
Invasive positive pressure ventilation	111 (3.0)	9 (3.6)	42 (3.0)	60 (2.9)	0.79
Renal replacement therapy	167 (4.4)	15 (5.9)	51 (3.6)	101 (4.7)	0.14
Pulmonary artery catheter	649 (17)	47 (19)	257 (18)	345 (16)	0.22
Intra-aortic balloon pumping	46 (1.2)	11 (4.4)	19 (1.4)	16 (0.8)	<0.001
Percutaneous cardiopulmonary support	10 (0.3)	1 (0.4)	3 (0.2)	6 (0.3)	0.85
**Invasive coronary evaluation and revascularization**					
Coronary angiography	1062 (28)	51 (20)	372 (26)	639 (30)	0.001
Percutaneous coronary intervention	217 (5.7)	9 (3.6)	80 (5.7)	128 (6.0)	0.29
Coronary artery bypass grafting	33 (0.9)	2 (0.8)	17 (1.2)	14 (0.7)	0.22

Values are number (%).

SBP=systolic blood pressure.
