# Supplementary material for: Admission systolic blood pressure as a prognostic predictor of acute decompensated heart failure: A report from the KCHF registry
Source: PLoS One. 2021 Jul 2;16(7):e0253999. doi: 10.1371/journal.pone.0253999 (PMC8253441; doi:10.1371/journal.pone.0253999)
Supplement: S3 Table — (PDF) [file pone.0253999.s005.pdf]

**S3 Table. In-hospital Clinical Outcomes.**

| Variables | Entire cohort (N=3804) | Admission SBP <100 mmHg (N=253) | Admission SBP 100-139 mmHg (N=1411) | Admission SBP ≥140 mmHg (N=2140) | P value |
|---|---|---|---|---|---|
| **In-hospital adverse events** | | | | | |
| Ventricular tachycardia or fibrillation | 164 (4.6) | 26 (11) | 66 (5.0) | 72 (3.6) | <0.001 |
| Ischemic stroke | 60 (1.6) | 1 (0.4) | 22 (1.6) | 37 (1.7) | 0.27 |
| Infection | 419 (11) | 33 (13) | 167 (12) | 219 (10) | 0.19 |
| Worsening renal function | 1224 (35) | 38 (19) | 376 (29) | 810 (40) | <0.001 |
| **Length of hospital stay, days** | 16 (11–24) | 17 (11–29) | 16 (12–27) | 15 (11–23) | <0.001 |

Values are number (%) or median (interquartile range).

Worsening renal function was defined as >0.3 mg/dL increase in serum creatinine during hospitalization.

Infection was defined as infection newly developed during hospitalization.

SBP=systolic blood pressure.
